# Hématomes calcifiés sous cutanées secondaires à des injections intra-musculaires

**DOI:** 10.11604/pamj.2018.29.199.12651

**Published:** 2018-04-06

**Authors:** Nehal Maja, Ouafa Hocar

**Affiliations:** 1 Faculté de Médecine et de Pharmacie, Université Cadi Ayyad Service de Dermatologie et Vénéréologie, CHU Mohammed VI, Marrakech, Maroc

**Keywords:** Hematoma, intramuscular injections, excision

## Image en médecine

Cutaneous and subcutaneous calcinoses are characterized by dermal or hypodermic depositions of hydroxyapatite, composed of phosphate ions and calcium. They are rare and must be distinguished from calcium deposits of the deep soft tissues. We report the case of a 85-year old female patient with a several year history of repeated intramuscular injections on a regular basis, probably due to mixed polyarthralgias. She presented with slightly hard plaques infiltrated into the two upper outer quadrant of the buttocks. The plaques were surmonted in the middle by calcified, roughly symmetrical nodules which were spontaneously painless and on palpation, evolving over the last twenty years without other associated signs. Laboratory tests were negative. The patient underwent skin biopsy which showed typical calcified hematoma appearance. The patient was referred to the Division of Plastic Surgery for possible excision.

Les calcinoses cutanées et sous cutanées sont caractérisées par des dépôts dermiques ou hypodermiques d'hydroxyapatite, composée d'ions phosphate et calcium. Elles sont rares et doivent être distinguées des dépôts calciques des tissus mous profonds. Nous rapportons le cas d'une patiente âgée de 85 ans, qui reçoit depuis plusieurs années des injections intra-musculaires répétées et à rythme régulier, probablement pour des polyarthralgies d'allure mixte qui s'est présenté en consultation pour deux placards légèrement indurés et infiltrés des deux quadrants supéro-externes des deux fesses, surmonté à leur centre par des nodules calcifiés, grossièrement symétriques et indolores spontanément et à la palpation, évoluant depuis une vingtaine d'années sans autres signes associés. Le bilan biologique réalisé est revenu négatif. La patiente a eu une biopsie cutanée ayant objectivé un aspect compatible avec un hématome calcifié. La patiente a été adressée en chirurgie plastique pour éventuelle excision.

**Figure 1:**
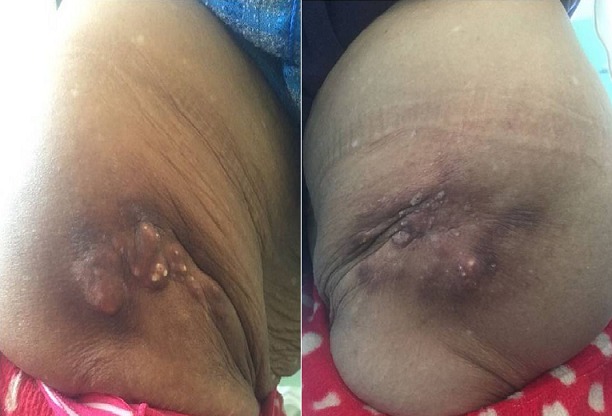
placards indurés et infiltrés bilatéraux et grossièrement symétriques de la racine des deux membres inférieurs, surmonté de nodules calcifiés à leur centre

